# Cytoophidia respond to nutrient stress in *Drosophila*

**DOI:** 10.1016/j.yexcr.2019.02.003

**Published:** 2019-03-15

**Authors:** Zheng Wu, Ji-Long Liu

**Affiliations:** a School of Life Science and Technology, ShanghaiTech University, 230 Haike Road, 201210 Shanghai, China; b MRC Functional Genomics Unit, Department of Physiology, Anatomy and Genetics, University of Oxford, South Parks Road, Oxford OX1 3PT, United Kingdom

**Keywords:** CTP synthase, Cytoophidium, *Drosophila*, Heat shock, Nutrient stress, Ring canal

## Abstract

CTP synthase (CTPsyn) is a metabolic enzyme essential for the de novo synthesis of CTP the nucleotide. CTPsyn can be compartmented into filamentous structures named cytoophidia. Cytoophidia are conserved in a wide range of species and are highly abundant in *Drosophila* ovaries. Here we report that cytoophidia elongate upon nutrient deprivation, CTPsyn overexpression or heat shock in *Drosophila* ovaries. We also show that the curvature of cytoophidia changes during apoptosis. Moreover, cytoophidia can be transported from nurse cells to the oocyte via ring canals. Our study demonstrates that cytoophidia can respond to stress and are very dynamic in *Drosophila* ovaries.

## 1 Introduction

CTP synthase (CTPsyn) is a critical metabolic enzyme that catalyzes the rate limiting step of de novo CTP synthesis pathway [1]. CTPsyn has been found to have the ability to form filamentous structures in various organisms [2], [3], [4], [5], [6], [7], [8]. Because of their serpentine morphology, these structures have been named as cytoophidia, meaning ‘cell snakes’ in Greek [3], [9], [10]. Previous studies demonstrate that CTPsyn form cytoophidia in response to stress or stimuli [11], [12], [13], [14].

*Drosophila* oogenesis is sensitive to nutrition stress, and poor nutrient conditions can induce apoptosis in early and mid- oogenesis [15]. In the ovary, somatic follicle cells surround 16 germline cells including an oocyte and 15 nurse cells that provide nutrition for the oocyte. These germline cells are connected via ring canals, which are cytoplasmic bridges composed of F-actin and associated proteins [16]. During oogenesis, nurse cells undergo multiple rounds of endocycles in which the cell replicates its DNA without division [17], [18]. As a result, nurse cells can acquire enormous cell size.

Cytoophidia are highly abundant in both germline and somatic cells in *Drosophila* ovaries [3]. A nurse cell usually contains one or a few long and thick cytoophidia (macro-cytoophidia) and numerous short and thin cytoophidia (micro-cytoophidia). The macro-cytoophidia in nurse cells during mid-oogenesis can reach 20–30 µm long. This makes nurse cells very suitable for studying cytoophidium dynamics and morphology.

Here, we report that starvation induces the elongation of cytoophidia during *Drosophila* mid-oogenesis in a reversible manner. Forks can be found on cytoophidia in starved egg chambers, which may represent the process of cytoophidia fusion during elongation. The straightness of cytoophidia decreases in the process of starvation induced mid-oogenesis apoptosis. Moreover, overexpressing CTPsyn in germline cells increases cytoophidum length and curvature. Cytoophidia can be transported from nurse cells to the oocyte via ring canals. Finally we find that cytoophidia also respond to heat shock. Our results show that cytoophidia are responsive to nutrient stress and heat shock, providing new insights for the stress coping function of cytoophidia.

## 2 Results

### 2.1 Nutrient deprivation induces cytoophidium elongation in nurse cells

**Fig. 1:**
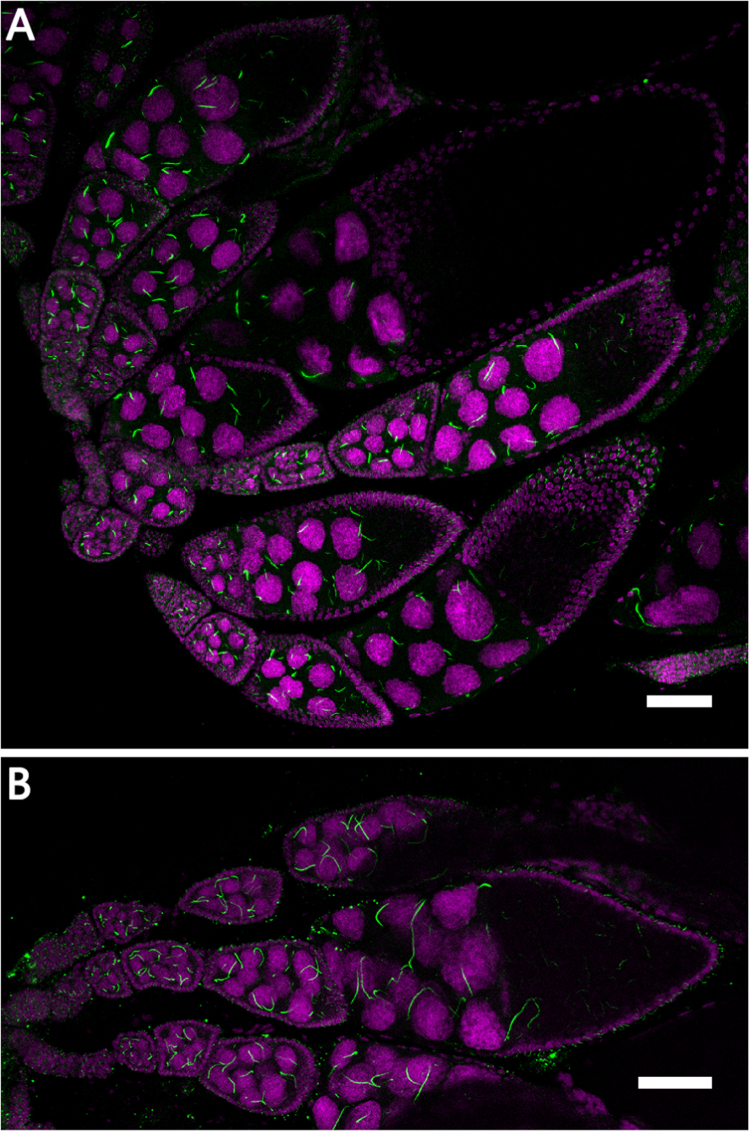
**Distribution of cytoophidia during early and mid- oogenesis.** Egg chambers from w^1118^ flies are stained with CTPsyn antibody (green). DNA is stained with Hoechst 33342 (magenta). (A) In a well-fed female, cytoophidia exists in nurse cells in early and mid-oogenesis. (B) In a starved female, cytoophidia in the early and mid-oogenesis become longer comparing to the well-fed fly. Scale bars, 50 µm.

**Fig. 2:**
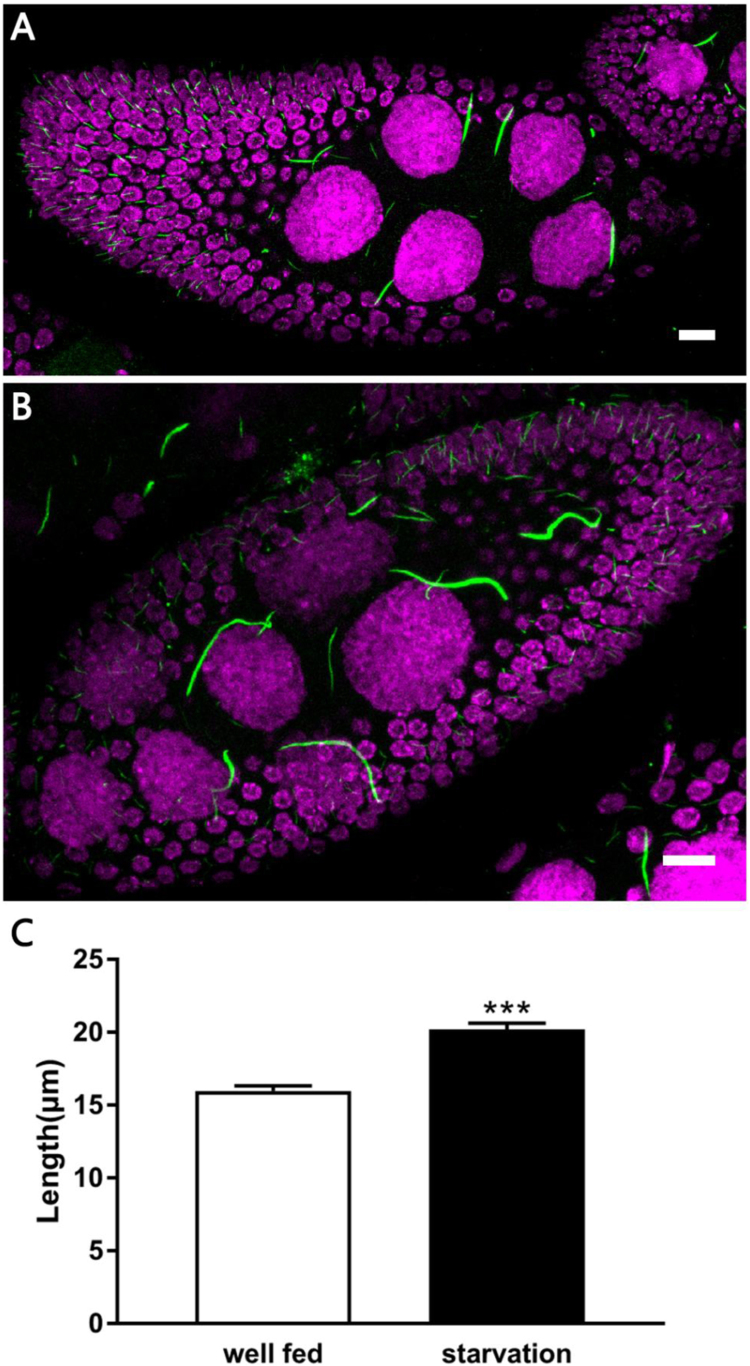
**Cytoophidia respond to starvation.** (A) Cytoophidia are shown in a stage 9 egg chamber from a well-fed female. (B) Cytoophidia are shown in a stage9 egg chamber from a starved female. (C) Cytoophidia show a significant increase in diameter in nutrient deprived conditions. DNA is labelled by Hoechst 33342 (Magenta in A and B). The mean Length of the cytoophidia in starved stage9 egg chambers is significantly longer than the mean Length of the cytoophidia in well-fed stage9 egg chambers. (Cytoophidia longer than 10 µm were counted, Data are mean ± SEM. *t*-test was used, p < 0.001, ***denotes statistical significance). Scale bars, 10 µm.

Cytoophidia can be detected in nurse cells during oogenesis (Fig. 1a). The length of cytoophidia in nurse cells increases as the egg chambers go through early and mid-oogenesis. Upon starvation, cytoophidia in nurse cells generally become longer (Fig. 1b). The elongation is obvious in mid-oogenesis (Fig. 2a, b). The length of cytoophidia in stage 9 egg chambers was measured to quantify the change of cytoophidia in response to nutrient deprivation. The mean length of cytoophidia in nurse cells was significantly greater in starved ovaries than that in well-fed ovaries in stage 9 egg chambers (P < 0.001) (Fig. 2c). These results suggest that cytoophidia respond to nutrient stress in the form of elongation.

### 2.2 Forks can be found on cytoophidia in starved egg chambers

Starvation induces cytoophidia elongation, yet the process of the elongation remains unknown., Most cytoophidia in nurse cells from well-fed females are smooth and do not interact with each other.

**Fig. 3:**
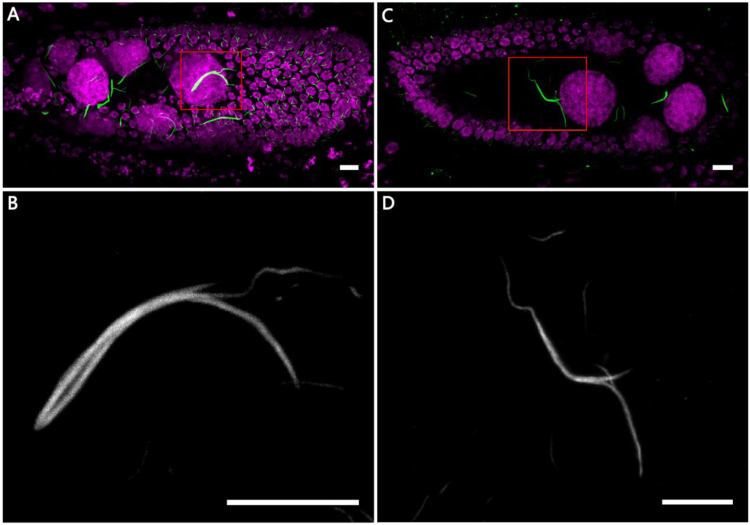
**Cytoophidia under starved condition.** (A, C) CTPsyn (green) staining of a stage 9 egg chamber from a starved female. DNA is labelled by Hoechst 33342. Note that forks can be detected on the cytoophidium highlighted by the red box. (B) Zoom in image of A. (D) Zoom in image of C. Scale bars, 10 µm.

However, upon starvation, forks can be found on cytoophidia (Fig. 3). Previous study has shown that cytoophidia can undergo fusions to become larger in mammalian cells [7]. These fork structures may represent the process of elongation, in which one cytoophidium fuses to another one.

### 2.3 Cytoophidia response to starvation is reversible

**Fig. 4:**
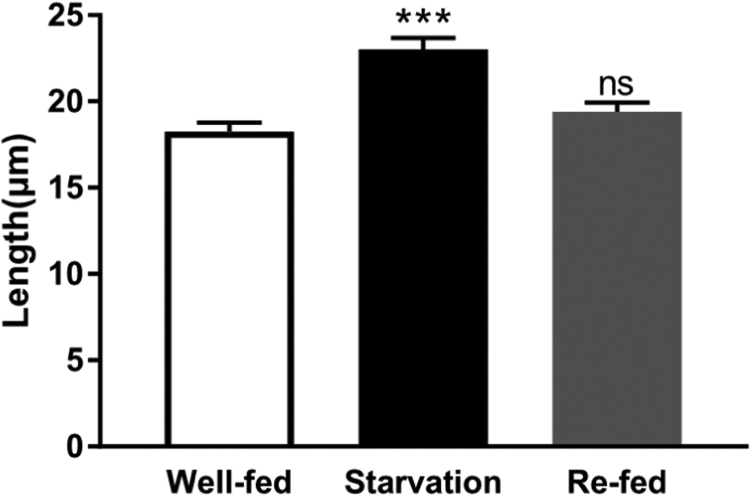
**Starvation induced cytoophidia elongation in stage 9 egg chambers is reversible.** The mean Length of cytoophidia increases upon starvation. After re-fed, there is no significant difference between the well-fed group and the re-fed group. Cytoophidia longer than 10 µm were counted, Data are mean ± SEM. *t*-test was used, p < 0.001, * **denotes statistical significance, n.s. denotes not significant.

During the neurogenesis of *Drosophila* larvae, starvation has been found to induce cytoophidia assembly in central nervous system, while re-feeding after starvation can induce cytoophidia dissociation. To test whether starvation induced cytoophidia elongation is reversible during oogenesis in adult flies, we analyzed cytoophidia length in stage 9 egg chambers from females that were re-fed after starvation. After re-fed, the length of cytoophidia decreased to the same level of the well-fed ones (Fig. 4). This suggests that the elongation of cytoophidia responding to nutrient condition is reversible.

### 2.4 Cytoophidia increase the curvature upon apoptosis

**Fig. 5:**
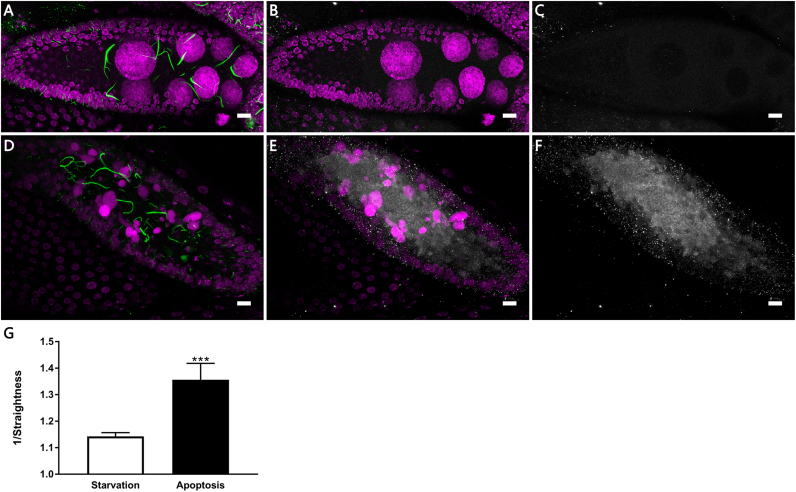
**The morphology change of cytoophidia in an apoptotic egg chamber during mid-oogenesis.** (A) Cytoophidia (green) in a non-apoptotic stage 9 egg chamber under starvation are generally straight. (B) The overlay image of DNA (magenta) and cleaved Caspase-3 (white) signal of the egg chamber in image A. (C) The signal from cleaved Caspase-3 (white) is very weak, showing that the egg chamber is not undergoing apoptosis. (D) Apoptosis in a stage 9 egg chamber is indicated by DNA (magenta) condensation. CTPsyn (green) staining shows cytoophidia in a very twisted form in the apoptotic egg chamber. (E) DNA (magenta) and cleaved Caspase-3 (white). (F) The strong signal from cleaved Caspase-3 (white) confirms that the egg chamber is undergoing apoptosis. (G) Cytoophidia are more twisted in apoptotic egg chambers.The reciprocal of straightness is used to show the significant change. DNA is labelled by Hoechst 33342 (Magenta in A and B). Data are mean ± SEM. ***P < 0.001. Scale bar, 10 µm.

**Fig. 6:**
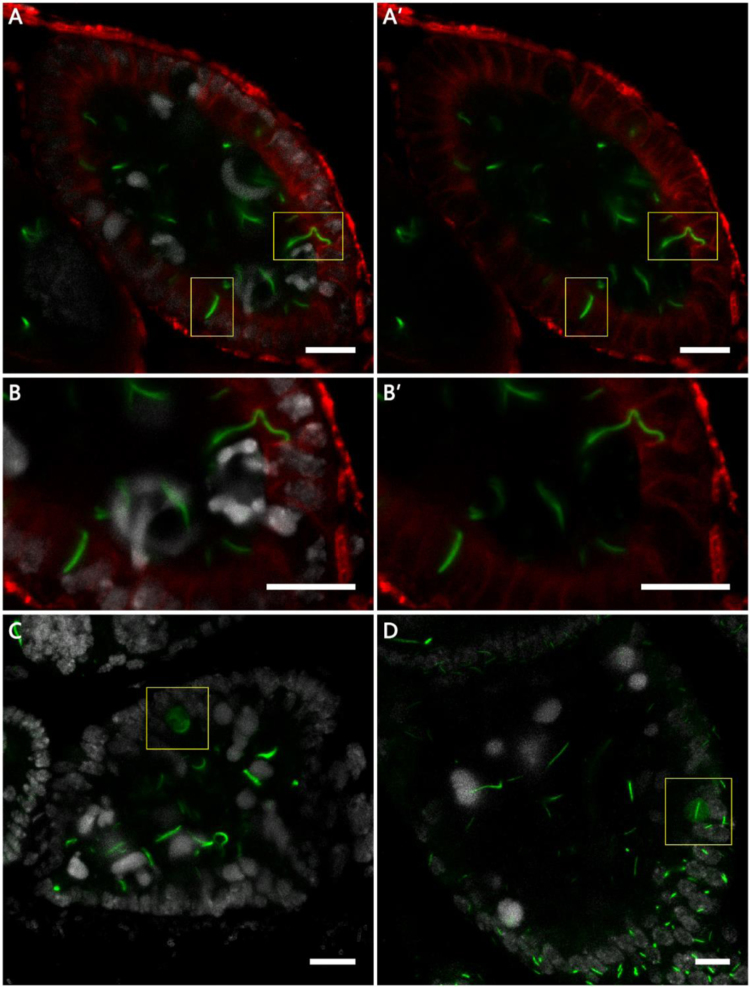
**The engulfment and degradation of cytoophidia by follicle cells.** DNA is labelled by Hoechst 33342 (white). (A and A′) Cytoophidia enter follicle cell during apoptosis (highlighted by the yellow box). The cell boundary is outlined by Discs Large (red). (B and B′) Zoom in of A and A′. (C and D)The diffused CTPsyn signal in follicle cell. Scale bars, 10 µm.

Poor nutrient condition can induce apoptosis during mid-oogenesis in *Drosophila* ovary [15]. Cytoophidia in apoptotic egg chambers have been reported to increase in number and are more likely to be entangled [3]. We analyzed the length and straightness of cytoophidia in stage 9 egg chambers undergoing apoptosis induced by starvation. Straightness is defined as the ratio of filament length and the distance between the starting and ending point of the filament (a straight filament's straightness is 1). In the early and mid-stage of apoptosis, there is no significant change in the length of cytoophidia, however there is a dramatic change in straightness (Fig. 5). During the process of apoptosis, the remnants of nurse cells are engulfed by surrounding follicle cells, and cytoophidia can be found entering the follicle cells (Fig. 6A and B). In the late stage of apoptosis, diffused CTPsyn signal with a round shape can be found in follicle cells (Fig. 6C and D), which may represent the disassembly process of cytoophidia. These findings may indicate that some intracellular components or microenvironments which are required to maintain cytoophidia morphology are degraded or disrupted during apoptosis before the degradation of cytoophidia.

### 2.5 Overexpressing CTPsyn increases the length and curvature of cytoophidia

**Fig. 7:**
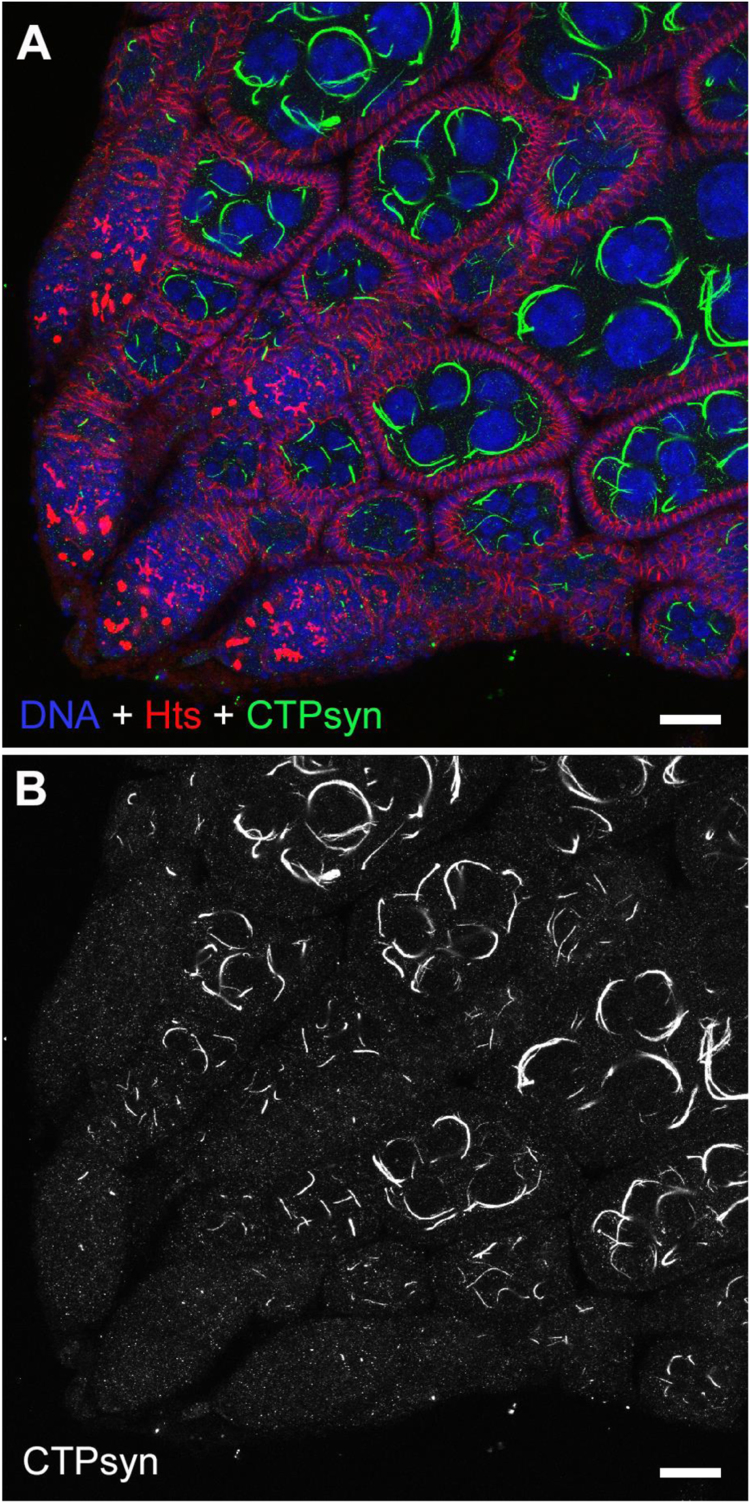
**Overexpressing CTPsyn increases the length and curvature of cytoophidia**. (A, B) Maternal Triple Drive (MTD-GAL4) induces germline-specific overexpression of CTPsyn (Green in A, white in B). The cell boundary is outlined by membrane protein Hu- li tai shao (Hts) (Red in A). DNA is labelled by Hoechst 33342 (blue in A). Note that cytoophidia in nurse cells and oocytes are elongated and much curved when CTPsyn is overexpressed (A, B), in comparison with those in wild-type flies with normal CTPsyn level (Fig. 1A). Scale bars, 20 µm.

Elongation of cytoophidia can also be induced by overexpressing CTPsyn, as we described previously [27]. Using a Maternal Triple Drive (MTD-GAL4), we can express UAS-CTPsyn-GFP specifically in the germline cells including nurse cells and oocytes in female reproductive system [27]. Consistent with our previous study, we observed that overexpressing CTPsyn increased the length and curature of cytoophidia in germline cells from early oogenesis to middle oogenesis (Fig. 7). Because MTD-GAL4 is strongly expressed in the germline, the overexpressed CTPsyn-GFP forms much longer cytoophidia than wild-type germline cells. While only one or a few macro-cytoophidia are detected in each nurse cell in wild-type flies, we observed many more macro-cytoophidia in nurse cells with high level of CTPsyn. Very often those elongated cytoophidia, with increased curvature, were tangled together surrounding the large nuclei of the nurse cells.

### 2.6 Cytoophidia are transported from nurse cells to the oocyte via ring canals

After four mitotic divisions, a germline cystoblast produces a 16-cell germline cyst. These divisions are atypical in that cytokinesis is incomplete, leading to cytoplasmic openings i.e. ring canals which bridge neighbouring germline cells. Among the 16 germline cells, one becomes the oocytes while the other 15 cells turn into nurse cells. As the name suggested, nurse cells produce nutrients including proteins, RNAs and organelles which are transported into the oocyte via ring canals.

**Fig. 8:**
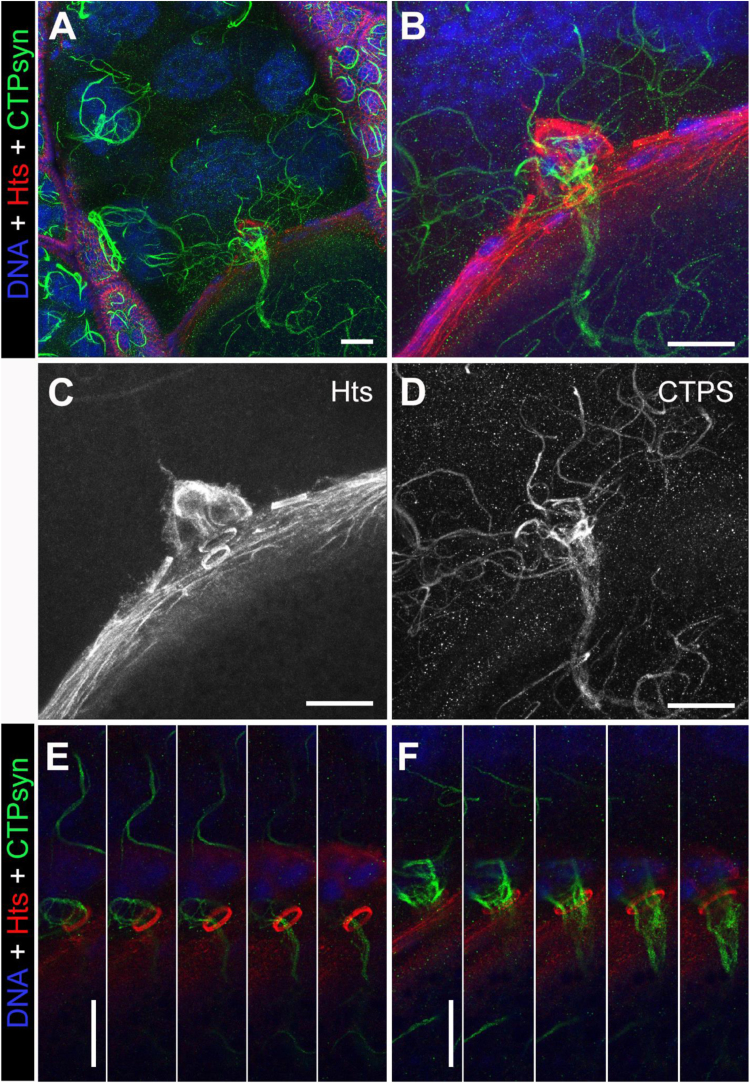
Cytoophidia are transported from nurse cells to the oocyte via ring canals when CTPsyn is overexpressed. (A, B) long and tangled cytoophidia induced by germline-specific overexpression of CTPsyn (green) are transported from nurse cells to the oocyte through four ring canals (rings labelled by Hts, red). B is a zoom-in image of A. (C, D) single-colored channels for Hts (C) and CTPsyn (D) of image B. (E, F) Cytoophidia travelling through two ring canals are captured by serial projections along the Z-axis. DNA is labelled by Hoechst 33342 (blue in A, B, E and F). Scale bars: 20 µm.

**Fig. 9:**
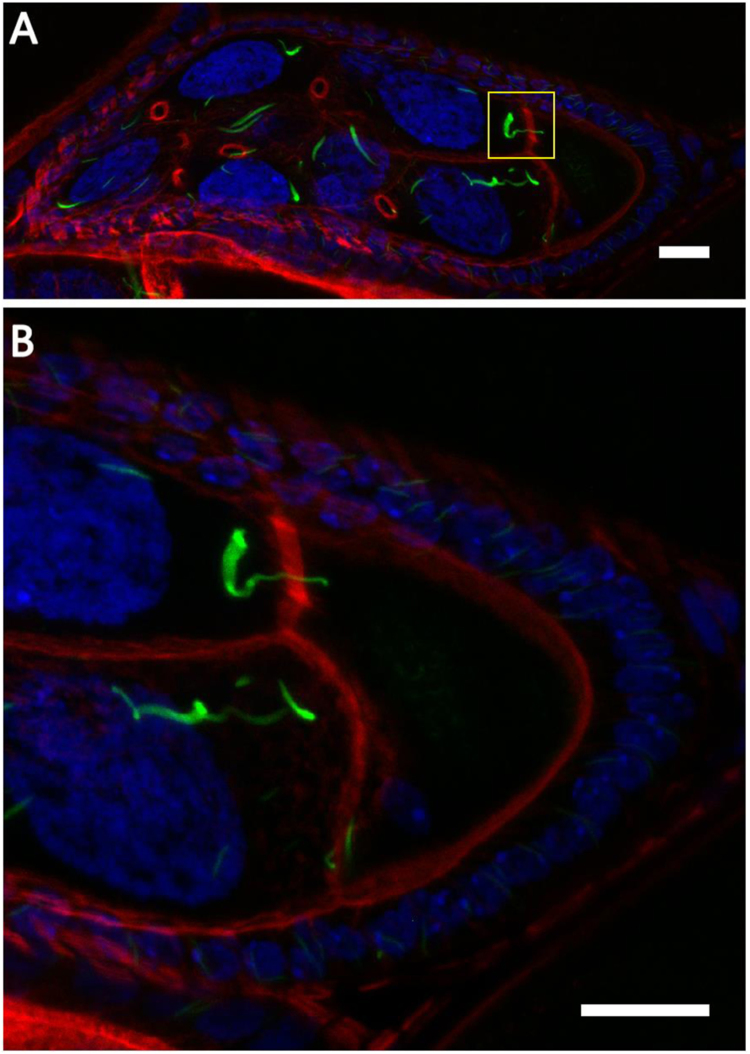
**Cytoophidia are transported from nurse cells to the oocyte via ring canals when CTPsyn level is normal.** (A) When CTPsyn level is normal, a cytoophidium is transported from nurse cell to the oocyte through a ring canal (highlighted by the yellow box). The rings are labelled by phalloidin (red). (B) Zoom in of A. DNA is labelled by Hoechst 33342 (blue). Scale bars: 10 µm.

To test if cytoophidia can be transported from nurse cells to the oocyte, we took advantage of the germline-specific expression of CTPsyn-GFP. Obvious and elongated cytoophidia can be observed throughout the cytoplasm of nurse cells as well as the ooplasm (Fig. 8A-D). Carefully examination of macro-cytoophidia at the border between nurse cells and the oocyte revealed that indeed macro-cytoophidia can travel from the nurse cells to the oocyte (Fig. 8E and F). Multiple macro-cytoophidia from the nurse cells can funnel through the ring canals, resulting in tightly bundled cytoophidia in the oocyte. When CTPsyn level is normal, we also observed that cytoophidia travel from nurse cells to the oocyte through the ring canals (Fig. 9).

### 2.7 Cytoophidia respond to heat shock

**Fig. 10:**
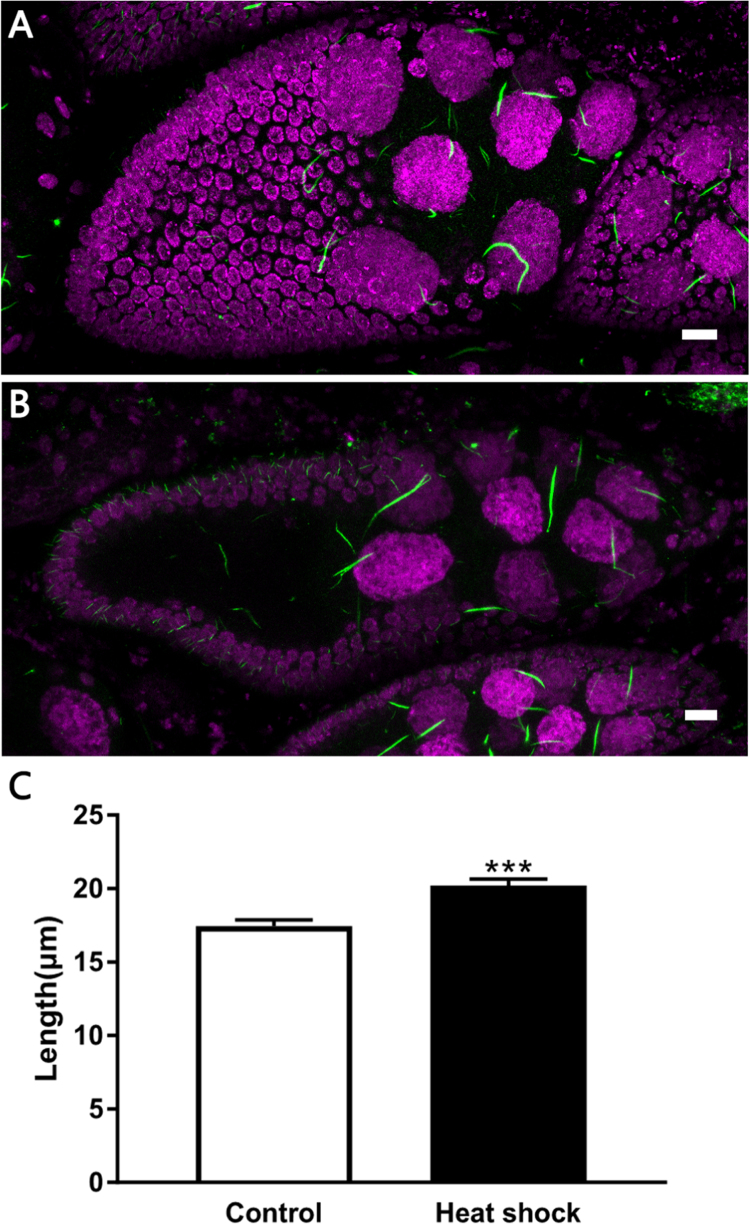
**Cytoophidia respond to heat shock.** (A) Cytoophidia (green) in a stage 9 egg chamber from a female that was kept in 25 °C for 1 h as control. (B) Cytoophidia (green) in a stage9 egg chamber from a female that was heat-shocked at 37 °C for 1 h. (C) Heat shock induces cytoophidia elongation. DNA is labelled by Hoechst 33342 (Magenta in A and B). Data are mean ± SEM. ***P < 0.001. Scale bars: 10 µm.

**Fig. 11:**
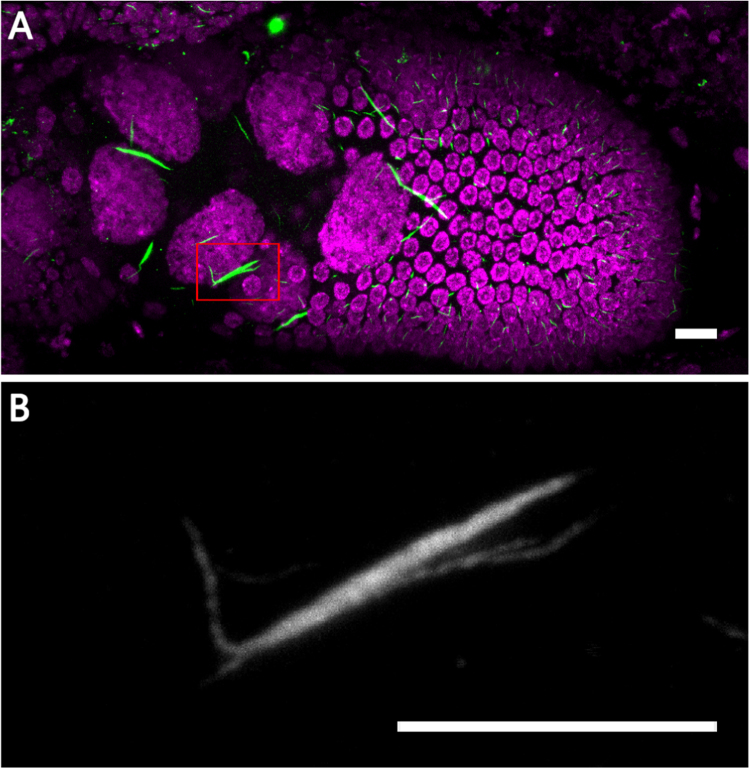
**Cytoophidia after heat shock.** (A) Cytoophidia (green) in a stage 9 egg chamber from a female after heat shock. DNA is labelled by Hoechst 33342 (Magenta). Note that forks can be found on the cytoophidium highlighted by the red box. (B) Zoom in image of A. Scale bars, 10 µm.

Heat shock has been shown to induce U bodies response in *Drosophila* ovary [19], so we test if heat stress can also induce cytoophidia response. We measured the length of cytoophidia in stage 9 egg chambers after heat shock. Cytoophidia became longer after being heat shocked at 37 °C for 1 h than those non-heat shock controls (Fig. 10). Forks can also be found on cytoophidia in egg chambers after heat shock (Fig. 11).

## 3 Discussion

We have demonstrated that cytoophidia elongate in response to nutrient stress in *Drosophila* ovaries. Previous studies have shown that CTPsyn assemble into cytoophidium in starved neuroblasts of the *Drosophila* larval central nervous system, and in nutrient deprived cultured cells which depends on histidine-mediated protein methylation [11], [14]. Consistently, the reversible elongation of cytoophidia in starved egg chambers provides further evidence supporting that cytoophidia are responsive to stress. The formation or elongation of cytoophidia may be a strategy to store CTPsyn molecules when the environment is not suitable for the organism to grow. When the environment gets better, CTPsyn molecules are released from cytoophidia, as indicated by the reversibility.

CTP plays an essential role in cell growth as a precursor in DNA and RNA synthesis. Besides, CTP is also involved in phospholipid synthesis [20]. Cytoophidia have been found to associate with Golgi bodies in germline cells of female flies [3]. During mid-stage oogenesis, neutral lipids accumulate in the egg chambers to serve as energy and material storage for vitellogenesis [21]. Considering these evidences together, cytoophidia elongation may be related to the lipid metabolism regulation in *Drosophila* female germline cells during nutrient stress. CdsA (CDP-diacylglycerol synthetase) catalyzes CTP and phosphatidic acid into CDP-diacylglycerol and has been found to play an important role in cell growth and fat storage [22]. Recently, a miRNA screen suggests that CdsA may be involved in the regulation of cytoophidia in *Drosophila* ovary [23]. This provides further evidence for the relation between CTPsyn and lipid metabolism.

The forks observed on the cytoophidia in egg chambers under stress suggest that the elongation may be accomplished by fusion. In cultured mammalian cells, small cytoophidia can fuse to form larger cytoophidia [7]. This indicates that fusion is a conserved strategy for Cytoophidia elongation in both vertebrates and in Invertebrates. Although the starvation induced elongation is reversible, the process of shortening remains unknown. Given that cytoophidia in egg chambers are large and responsive to stress, it will be interesting to study the mechanism of cytoophidia elongation and shortening by living imaging of egg chambers.

In the process of starvation induced apoptosis, the straightness of cytoophidia decreases dramatically. In healthy cells, caspases are expressed as inactive zymogens. During apoptosis, caspases are cleaved into active form and begin to degrade proteins. Effector caspase Dcp-1 plays a major role in the process of mid-oogenesis apoptosis [24]. In addition, the proform (zymogen) of Dcp-1 has been shown to regulate mitochondrial dynamics in *Drosophila* [25]. The consistency in time of caspase activation and cytoophidia straightness reduction suggests that caspase also regulate the morphology of cytoophidia directly or indirectly.

Due to their large size, germline cells in *Drosophila* female reproductive system have been attractive model systems to study various biological processes [17]. From nurse cells to the oocyte, nutrients and subcellular organelles are transported via ring canals [16]. Before this study, we do not know if the cytoophidium, a novel type of membraneless organelle, can travel through the ring canal among neighbouring germline cells. Interestingly, we were able to capture the very moment when cytoophidia go through a ring canal. Why does CTPsyn form visible structures under light microscopy and travel intercellularly among germline cells? One possible explanation is that cytoophidia sequester enzymatic activity [11], [12], [13], [26]. The transport of cytoophidia from nurse cells and the oocyte enables sufficient deposit of the sequestered enzyme into the mature oocyte, which in turn provides necessary materials for the development of early embryos.

In conclusion, our study describes that stress can induce reversible elongation of cytoophidia in *Drosophila*, and apoptosis can decrease the straightness of cytoophidia. These results indicate that the regulation of cytoophidia dynamics and morphology is related to stress coping. In addition, cytoophidia can be transported from the nurse cells into the oocyte via ring canals. Further study of the mechanism of cytoophidia regulation under stressed conditions is important for a better understanding of the function of this conserved filamentous organelle.

## 4 Materials and methods

### 4.1 Fly stocks and starvation assay

Flies were raised at 25 °C on standard cornmeal diet. *w*^*1118*^ was used as wild-type. Flies at the same age were starved in vials containing agar gel as water supply. Flies were starved for 6 h before ovaries were dissected. Control group were fed on standard cornmeal diet. In re-feeding experiment, flies were starved for 6 h and then re-fed on standard cornmeal diet for 24 h before dissection.

### 4.2 CTPsyn overexpression

Transgenic flies expressing full length CTPsyn (isoform C, LD25005) under the UAS promoter (UAS-CTPsyn) was generated in the lab previously [27]. To generate germline cells overexpressing CTPsyn, maternal triple drive (MTD-GAL4) virgin female flies were crossed to UAS-CTPsyn male flies. F1 flies were collected for tissue dissection.

### 4.3 Heat shock

Well-fed adult flies at the same age in empty vials were incubated in 37 °C water bath for 1 h. Control group in empty vials were kept at 25 °C for 1 h. After heat shock, ovaries were dissected immediately.

### 4.4 Immunofluorescence

Ovaries were dissected in Grace's Insect Medium, fixed in 4% paraformaldehyde in PBS for 10 min and washed with PBT (1X PBS + 0.5% horse serum + 0.3% Triton X-100). Tissues were incubated in primary antibodies at room temperature overnight, washed with PBT, and then incubated at room temperature overnight in secondary antibodies. Primary antibodies used in this study included rabbit anti-CTPsyn (1:1000; y-88, sc-134457, Santa Cruz BioTech Ltd, Santa Cruz, CA, USA), goat anti-CTPsyn (1:1000; Santa Cruz 33304), rabbit anti-Caspase-3 (1:500; Abcam ab13847), mouse anti-Hu-li tao shao (Hts) (1:20; 7H9 1B1, Developmental Studies Hybridoma Bank, Iowa City, IA, USA) and rabbit anti-Discs Large. Secondary antibodies used in this study were anti-mouse, rabbit, or goat antibodies that were labelled with Alexa Fluor® 488 (Molecular Probes), or with Cy5 or Dylight 649 (Jackson ImmunoResearch Laboratories, Inc.). Fluorescence labelled phalliodin was used to label actin filaments. Hoechst 33342 were used to label DNA.

### 4.5 Confocal microscopy and image analysis

All images were obtained using confocal microscopes (Leica TCS SP5II or Leica SP8, Leica Microsystems CMS GmbH, Mannheim, Germany). Image processing was conducted using Leica Application Suite X. Imaris 9 was used to analyze the length and straightness of cytoophidia. For each condition, at least eighteen egg chambers were analyzed. Cytoophidia longer than 10 µm were selected for calculation. *t-*test was used to check for significant difference. Significant differences were attributed for p < 0.05.
